# Gender difference in spontaneous deception: A hyperscanning study using functional near-infrared spectroscopy

**DOI:** 10.1038/s41598-017-06764-1

**Published:** 2017-08-08

**Authors:** Mingming Zhang, Tao Liu, Matthew Pelowski, Dongchuan Yu

**Affiliations:** 1 Key Laboratory of Child Development and Learning Science, Ministry of Education, Southeast University, Nanjing, 210096 China; 2 Research Center For Learning Science, School of Biological Science & Medical Engineering, Southeast University, Nanjing, 210096 China; 3 Department of Marketing, School of Management, Zhejiang University, Hangzhou, 310058 China; 4 Department of Basic Psychological Research and Research Methods, Faculty of Psychology, University of Vienna, Vienna, Liebiggasse 5 1010 Austria

## Abstract

Previous studies have demonstrated that the neural basis of deception involves a network of regions including the medial frontal cortex (MFC), superior temporal sulcus (STS), temporo-parietal junction (TPJ), etc. However, to test the actual activity of the brain in the act of deceptive practice itself, existing studies have mainly adopted paradigms of passive deception, where participants are told to lie in certain conditions, and have focused on intra-brain mechanisms in single participants. In order to examine the neural substrates underlying more natural, spontaneous deception in real social interactions, the present study employed a functional near-infrared spectroscopy (fNIRS) hyperscanning technique to simultaneously measure pairs of participants’ fronto-temporal activations in a two-person gambling card-game. We demonstrated higher TPJ activation in deceptive compared to honest acts. Analysis of participants’ inter-brain correlation further revealed that the STS is uniquely involved in deception but not in honesty, especially in females. These results suggest that the STS may play a critical role in spontaneous deception due to mentalizing requirements relating to modulating opponents’ thoughts. To our knowledge, this study was the first to investigate such inter-brain correlates of deception in real face-to-face interactions, and thus is hoped to provide a new path for future complex social behavior research.

## Introduction

Deception, in psychology, is defined as a mental process through which an individual deliberately convinces others to accept a false belief in order to gain some type of benefit or to avoid loss for the deceiver^1^. Similarly, from an economic viewpoint, deception is defined as “a successful or unsuccessful deliberate attempt, without forewarning, to create in another [an untrue] belief in order to increase the communicator’s payoff at the expense of the other”^2^. Because of its importance in human development and social behaviors, deception has received a good deal of empirical interest. Various experimental paradigms have been used to investigate the behavioral and neural mechanisms of deception. Behavioral studies have, for example, consistently demonstrated a longer reaction time in deceptive than in honest acts. Similarly, neuroimaging studies have revealed that deception involves complex executive functions, identified through heightened brain activation in multiple areas when we deceive^1, 3–10^.

Sip (2008) notably proposed that deception mainly consists of three cognitive processes: (1) mentalizing, (2) decision making (including risk taking and reward processing), and (3) behavioral inhibition and control^11^. The neural mechanisms underlying these cognitive processes have been examined separately by a large number of studies^12–21^. Mentalizing is an ability to read and modulate mental states of others, in particular their intentions and beliefs, also known as “theory of mind”^15^. Mentalizing involves an extended brain network, including the dorsomedial and ventromedial prefrontal cortex (dmPFC and vmPFC), the dorsal anterior cingulate cortex (dACC), the posterior cingulate cortex (PCC), the posterior superior temporal sulcus (pSTS), and the temporo-parietal junction (TPJ)^22^. Concerning decision making in deception, Sip and colleagues (2012) revealed that the caudate and the inferior frontal gyrus (IFG) are also closely associated with expected reward and risk avoidance. Furthermore, they speculated that the ACC, the caudate, and the IFG play critical roles in mediating a decision to deceive based on context^11^. The dorsolateral prefrontal cortex (dlPFC), IFG, and the dACC are finally main regions involved in behavioral inhibition^19, 20^.

At the same time, although deception has been well examined by these studies, most have adopted passive deception paradigms, which tend to omit or minimize the above-mentioned spontaneity and internally-generated decisiveness aspects. Typically, in pursuit of a controlled task, participants are told to lie intentionally within certain blocks, allowing for comparison against a control (non-lie) task. However again, the definition of deception reveals two main critical characteristics: (1) it must be intentional. That is, it should occur as a decision from an individual without being forced from an external agent^23^. (2) It is generated spontaneously without early warning as part of an ongoing interaction with another participant^24^. Equally important, because of the confines of a scanner, studies typically focus on individual participants, often interacting with others via a monitor. Deception in our real life is however a dynamic, social, interactive process, involving the interplay between individuals often conversing face-to-face. We may employ language as well as numerous nonverbal social and cognitive skills, requiring the coordination of behavior according to a shared set of rules and customs, which may be lost in a scanner task^25^. This of course raises the question of the ecological validity of current results, or whether findings or activated areas might differ in other cases.

One final limiting aspect for previous studies involves a focus mainly on the individual brains of participants (see Hasson *et al*., 2012 for review)^25^. This omits the potential for uncovering online adaption or even coupling in activations between interactants. Recently it has been argued that, just as individuals’ bodies may intact in an interpersonal space, a complete understanding of the cognitive processes within a single individual’s brain cannot be achieved without examining and understanding the interactions with other humans^26^. Therefore, in order to better understand the roots of deception itself, it is necessary to assess both more natural human-human interactions and, simultaneously, reciprocal activations in the brains of two or more participants^25, 27^.

Such an ecologically valid, interactive approach—as we will employ in the present study—can be accomplished through a new technique called “hyperscanning”^28^, which has shown important promise for interactive studies in social neuroscience. This employs brain imaging, such as with functional magnetic resonance imaging (fMRI), electroencephalography (EEG), or functional near-infrared spectroscopy (fNIRS), to simultaneously measure neural activities of multiple individuals in shared tasks (for review, see Liu & Pelowski)^29^. This not only allows for the online monitoring of interactions between two individuals’ brains, especially the latter two imaging approaches (fNIRS and EEG) also allow individuals to share the same room and face-to-face setting. Hyperscanning techniques have shown promise in uncovering the behavioral and neural mechanisms of social decision-making behaviors in natural environments. Studies have investigated the Prisoner’s dilemma game^30^, the ultimatum game, and other card gambling games played by more than two participants^31^, as well as social discussions^32^, or simultaneous playing of musical instrument by musician groups^33^. These studies have both confirmed increased coherence in activations of paired participants in frontal regions and TPJ. Similar to the literature on deception, studies have also found larger activity in prefrontal areas under cooperation conditions in the Prisoner’s dilemma game^31^ and right temporo-parietal junction (rTPJ) in face-to-face ultimatum games^34^. In addition to results that are consistent with the findings obtained using conventional recording techniques, studies have also added specific information about the dynamics of social exchanges^35^, for example, regarding interpersonal synchronizations in social information exchanges^36^. If using fNIRS or EEG, we can finally consider the *temporal* dynamics of interaction at the resolution of milliseconds to a few seconds, allowing analysis of correlation between activation, or directionality of activation flows, in brains of both participants. For example, researchers have used key-press and finger-movement tasks to investigate cooperative behavior, showing increased coherence in right superior frontal cortices^37^ and right centroparietal cortex during cooperation^38^. These results suggest both the promise of hyperscanning in general, and also suggest great promise for deception analysis, with many of the same findings expected here as well.

The present study aimed to examine neural substrates underlying spontaneous deception in a two-person, gambling card-game task using the technique of hyperscanning employing fNIRS. Pairs of participants (always same M-M or F-F gender) took different roles, alternating between rounds, and played the game in a turn-taking style using a common money pot with the incentive of pocketing their bets for themselves. One participant (the ‘banker’) was instructed to look at his/her card and to bet. The other (the ‘follower’) was asked to call or not according to the banker’s bet without checking his/her own card. If the follower decided to call (expecting that the betting jetton was higher than or equal to the other player’s jetton), both would uncover their cards with the winner being the individual with the higher number, and again allowed to pocket the bet money for themselves. If the follower refused to call, the banker automatically won and pocketed their own bet. This paradigm thus gave the banker the incentive to bluff or deceive the other player in order to minimize losses and/or draw out the follower’s own bet. Likewise, the follower could try to ascertain if their partner was bluffing within the game.

Following previous studies on deception^39^, we measured participant pairs’ fronto-temporal cortices as the region of interest (ROI). We had the general hypothesis that, as shown in previous single brain studies, when the assigned “banker” bluffed or attempted to deceive we would uncover higher activations in the mPFC, dlPFC, TPJ, and STS. Through the use of hyperscanning, the study also allowed us to consider two further hypotheses concerning interaction between participants’ brains: First, since mentalizing is argued to be a core process in deception, participant pairs, throughout the unfolding game, were expected to potentially reach a shared representation about each other’s beliefs and intentions, and in turn to show synchronized activations between their brains. This followed studies concerning competition that similarly showed interpersonal synchronization in a competitive “placing disks” game^40^, and in our study would concern both the deceiver and the deceived. Second, previous studies have demonstrated that males and females may show different behavioral performance in deception frequency and reaction time^41^. Some studies have revealed that males may generate more deception than females (e.g., in academic dishonesty studies in which participants were invited to cheat by confederates and based on provided self-reports of cheating^42–45^). However, to date such gender differences had not been assessed in ecological valid, real life social neuroscanning assessments. Thus we were interested in the question of whether genders would show different patterns of inter-brain couplings in deception as well.

## Results

### Behavioral data

The behavioral results demonstrated that deception was a common tactic across conditions of the card game task. The deception rate (as assessed via procedure described in Methods, Data Analysis below) for all trials was 53.08% for males, and 54.58% for females. To examine the effect of gender on deceptive behavior, we conducted a 2 × 2 ANOVA with gender (male vs. female) as a between-participant factor and behavior-type (deception vs. honesty) as a within-participant factor. The dependent variable was the frequency of behavior-type (deception vs. honesty). The analysis revealed no main effects for gender [*F*(1,56) = 0, *p* > 0.05] or for behavior-type [*F*(1,56) = 0.836, *p* = 0.363], and no significant interaction [*F*(1,56) = 0.120, *p* = 0.730]. The frequency of deception was Mean = 8.08 [SD = ±2.452]; frequency of honesty was M = 6.91 [±2.452]. In addition to suggested equivalence in frequency of deceptive/honest behavior between genders, the above results thus also suggested that participants understood the game protocol and could sponteneously generate deception depending on the perceived monetary benefit.

**Figure 1:**
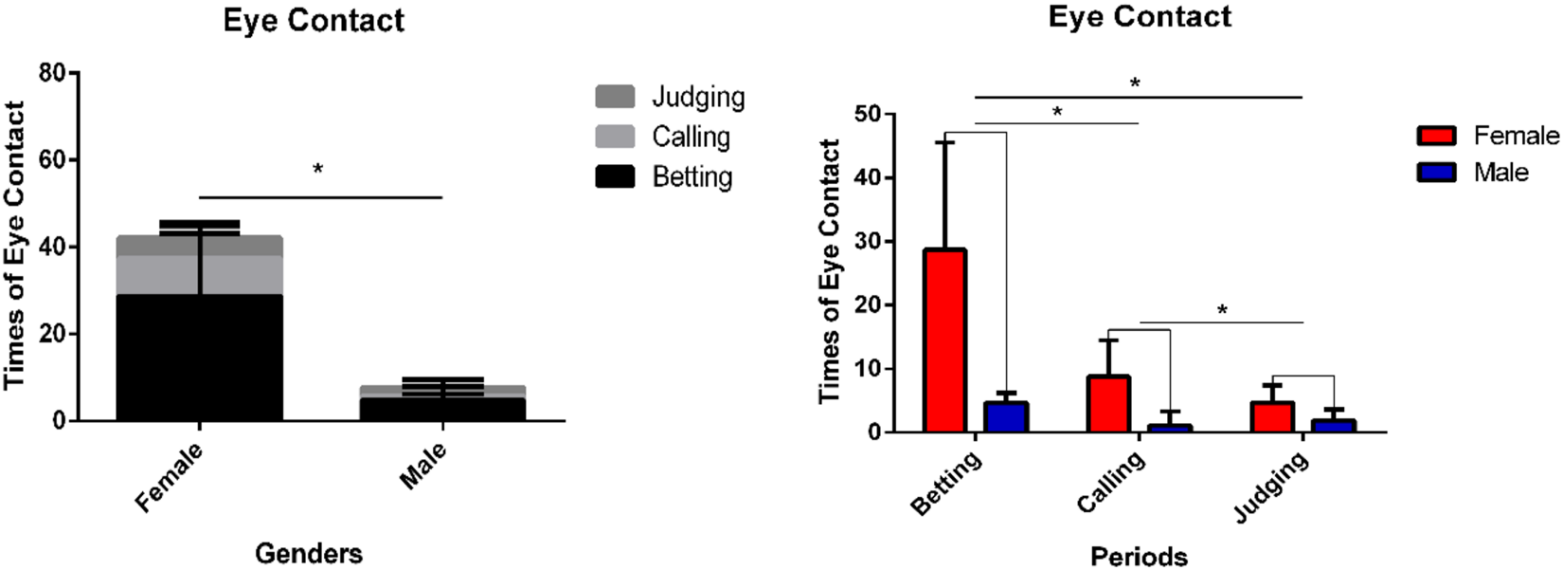
Eye contact between participant dyads. The gender difference of times of eye contact (left) and difference in times of eye contact between three stages (right). (*P < 0.05) Error bars indicate standard errors. (FDR corrected).

In addition, we assessed eye contact between participant dyads. Eye contact, among other ecological aspects related to human-to-human interaction, has previously shown significant gender differences—i.e., males may perceive eye contact, considered an indicator of ‘*mutual-communication’* within the game and recorded by video, as more threatening than females^46^. Transversely, females may use eye contact more and notice the loss of visual contact more than males^47^. Thus, the number of eye contacts between participant dyads was counted and analyzed via 2 × 2 ANOVA with gender (male vs. female) as a between-participant factor and the three stages (banker betting, follower calling, judging period) as a within-participant factor. This revealed significant main effects of gender [*F*(1,16) = 18.696, *p* = 0.001, ${\eta }_{p}^{2}$ = 0.539] and stages [*F* (2,32) = 26.474, *p* < 0.001, ${\eta }_{p}^{2}$ = 0.623], and a significant interaction effect [*F* (2,32) = 15.354, *p* < 0.001, ${\eta }_{p}^{2}$ = 0.490]. A simple effect test found that in the male dyads, the betting stage generated more eye contact than calling stages [*t*(8) = 4.257, *p* = 0.006, d = 3.010, Bonferroni adjusted] and judging stages[*t*(8) = 3.594, *p* = 0.018, d = 2.541, Bonferroni adjusted]. Comparison of calling stages and judging stages showed no difference [*t*(8) = −1.986, *p* = 0.087, d = 1.995]. In the female dyads, the betting stage generated more eye contact than calling [*t*(8) = 4.391, *p* = 0.004, d = 3.105, Bonferroni adjusted] and judging stages [*t*(8) = 4.865, *p* = 0.002, d = 3.440, Bonferroni adjusted]. The calling stage also generated more eye contacts than the judging stage [*t*(8) = 2.821, *p* = 0.044, d = 1.995, Bonferroni adjusted]. Comparison between genders within each stage also revealed that in both the betting [*t*(8) = 4.084, *p* = 0.008, d = 2.888, Bonferroni adjusted] and calling [*t*(8) = 4.082, *p* = 0.008, d = 2.886, Bonferroni adjusted] stages females generated more eye contacts than males [the judging stage showed no eye contact difference *t*(8) = 2.294, *p* = 0.051, d = 1.622]. Comparison of eye contact between bankers and followers showed no significant difference in all three stages: betting [*t*(17) = −1.186, *p* = 0.252, d = −0.575]; calling [*t*(17) = 1.317, *p* = 0.205, d = 0.639]; judging [*t*(17) = −0.223, *p* = 0.826, d = −0.108] (See Fig. 1).

### The fNIRS data: Deception versus honesty with gender difference

In order to guarantee the validity of the results, only pairs who deceived more than 10 times (9 male pairs and 9 female pairs; 18 out of 29 total pairs), were analyzed. According to the game procedure, each game round was divided into three periods: betting, follower calling, and judging (see also Methods below). Brain activations between the deception and honesty behaviors were compared in these three periods separately.

To examine activation differences between the deception and honesty behaviors in male and female “banker” participants, we conducted a two-way ANOVA [Gender (male vs. female) × behavioral-type (deception vs. honesty)] on Oxy-Hb in each channel. In the banker betting period, the ANOVA revealed no main effect of gender, but a significant main effect of behavioral-type in channels 4 and 23, corresponding to the mPFC [*F*(1,16) = 5.38, *p* = 0.030, ${\eta }_{p}^{2}$ = 0.262], as well as in channels 15 and 34 [*F*(1,16) = 4.975, *p* = 0.040, ${\eta }_{p}^{2}$ = 0.237], 16 and 35 [*F*(1,16) = 10.186, *p* = 0.006, ${\eta }_{p}^{2}$ = 0.389], and 17 and 36 [*F*(1,16) = 13.297, *p* = 0.002, ${\eta }_{p}^{2}$ = 0.454]. However, only channels 16/35 and 17/36 (mainly covering the temporal parietal junction, TPJ) survived after FDR correction (*p* < 0.05)]. A simple main effect test revealed that deception showed significantly higher activation than honesty (channels 16/35, *p* = 0.006; channels 17/36, *p* = 0.002). There were no significant interactions of gender by behavioral-type in all ROIs.

**Figure 2:**
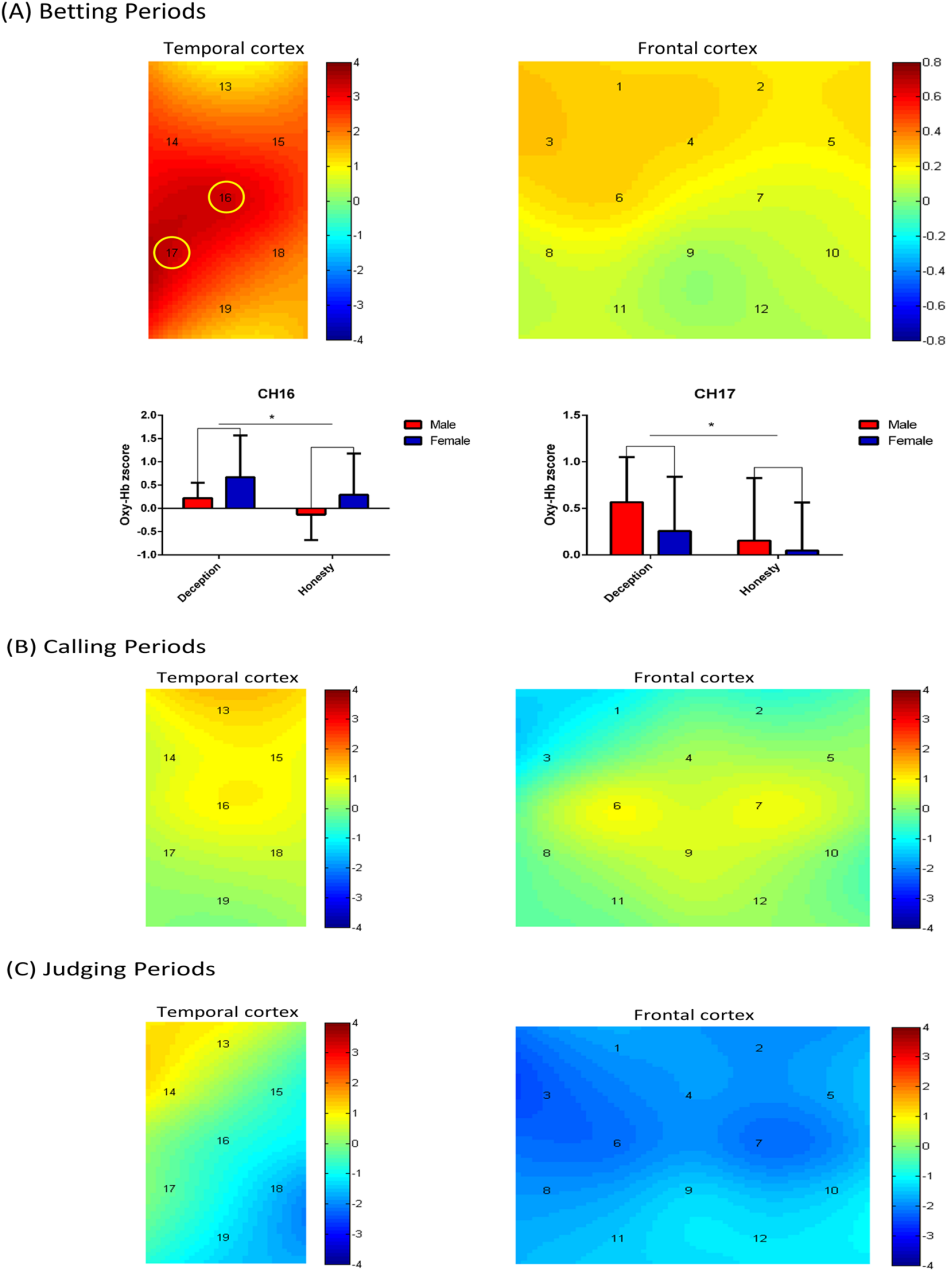
One-sample t-test of banker’s Oxy-Hb changes (deception compared with honesty) in (**A**) “banker” betting stage, (**B**) “follower” calling stage, and (**C**) judging stage, respectively. In the (A) “banker” betting stage, CH16 and CH17 (mainly covering TPJ) showed significantly higher activation in deception conditions (*P < 0.05). Error bars indicate standard errors. (FDR corrected). [Color bar represent t value].

In the follower calling period there were no main effects of gender and behavioral-type and no significant interaction in all channels. In the judging period, the ANOVA also revealed no main effect of behavior-type in all channels, but a significant main effect of gender in channels 4 and 23 (mPFC)[*F*(1,16) = 6.04, *p* = 0.026, ${\eta }_{p}^{2}$ = 0.27] and in channels 19 and 38 (superior temporal sulcus, pSTS)[*F*(1,16) = 4.51, *p* = 0.05, ${\eta }_{p}^{2}$ = 0.22]. However, no significant main effect of gender was detected in any channels after FDR correction (*p* < 0.05). The simple main effect test revealed that the neural activation of males was higher than females in these channels (See in Fig. 2). There was no significant interaction of gender by behavioral-type.

### Inter-brain correlations between banker-follower pairs

#### Interpersonal neural synchronizations (INS)

**Figure 3:**
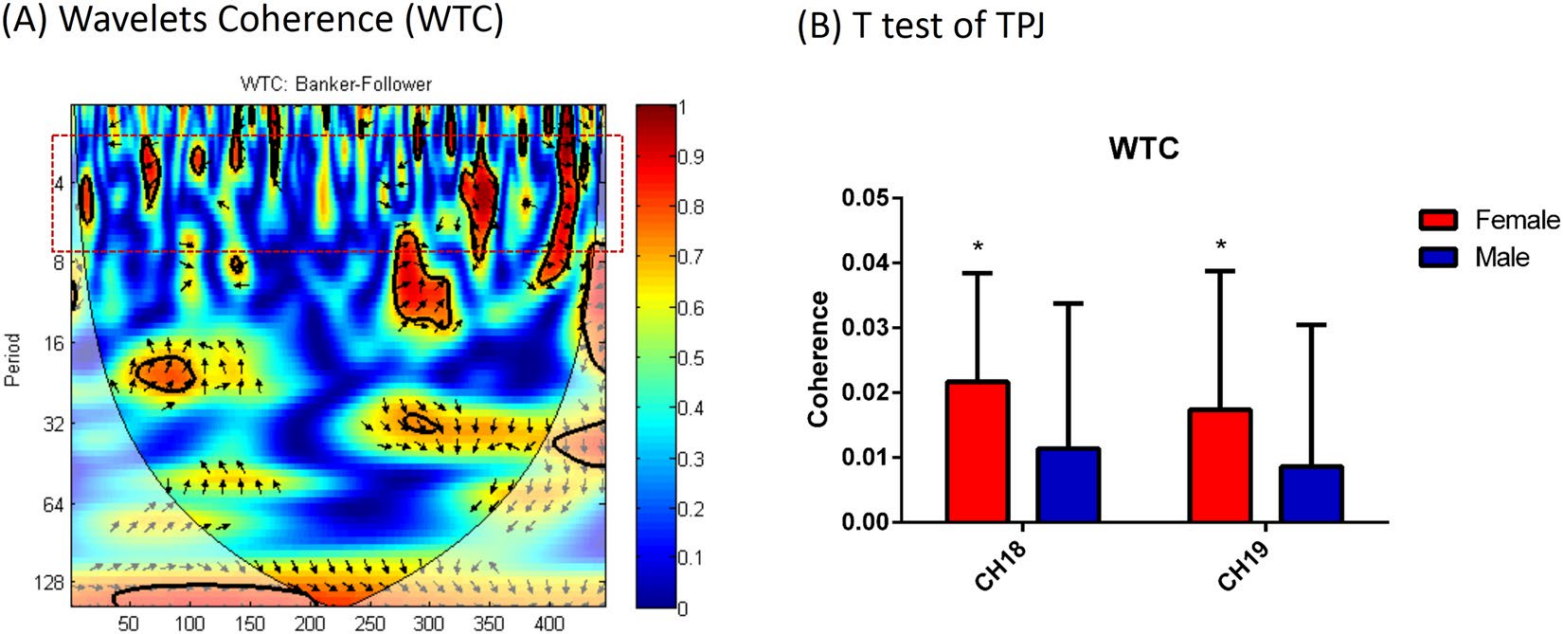
(**A**) Example of synchronization indicated by coherence. Wavelet transform coherence (WTC) based on oxy-Hb signal from channel 18 from banker and follower in the same dyad. The higher coherence encoded by red is in the task frequency band (2–7 s). (**B**) One-sample t-test of interpersonal brain synchronization in CH18 and CH19, respectively. Female banker–follower dyads showed significant synchronization in these two channels (*P < 0.05). Error bars indicate standard errors. (FDR corrected).

To analyze inter-brain correlations, we assessed *interpersonal neural synchronizations* (INS). This provides a measure of “coherence” between signals in pairs of brains engaged in an interactive task. We followed Cui (2012) to define“coherence increase” as the average coherence value in the deception blocks, minus the honesty blocks (see also detailed discussion in Analysis below)^37^. For each channel, a one-sample t-test of coherence increase across all participant dyads revealed significantly higher increases in the band between 0.05 and 0.2 Hz in CH18 (*t*(17) = 3.528, *p* = 0.003) and in CH19 (*t*(17) = 2.93, *p* = 0.020) (FDR corrected), corresponding to the pSTS. Independent samples t-test comparisons for each gender group further revealed only significant interpersonal neural synchronizations in the female dyads [CH18 (*t*(8) = 3.880, *p* = 0.005) and CH19 (*t*(17) = 2.450, *p* = 0.040) (FDR corrected)], but not in the male dyads [CH18 (*t*(8) = 1.529, *p* = 0.165) and CH19 (*t*(17) = 1.178, *p* = 0.273)(FDR corrected)] (See in Fig. 3).

#### The INS-Behavior Relation

**Figure 4:**
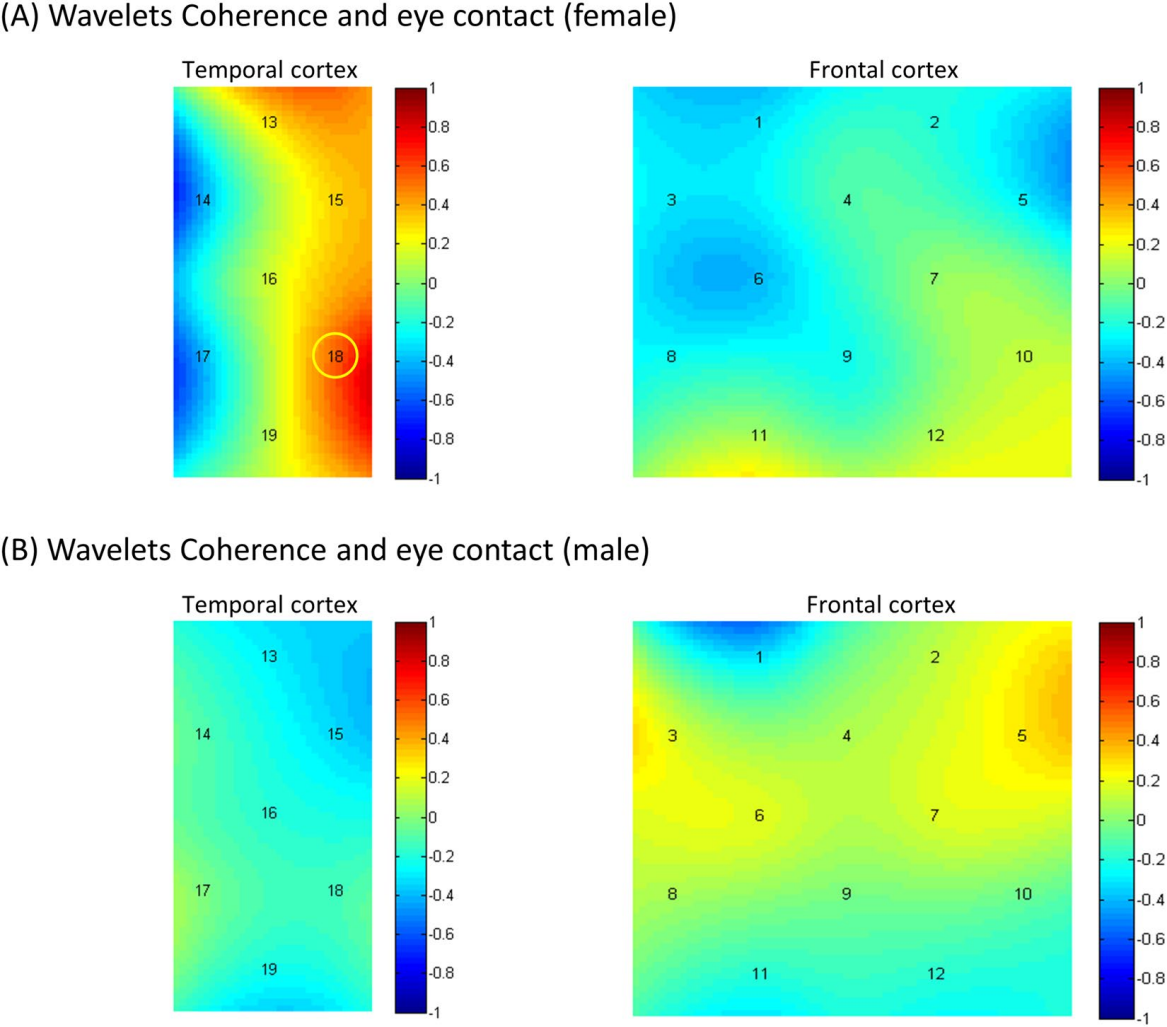
The Pearson correlation analyses of INS-Behavior. In all dyads, the deception INS and times of eye contact were positively correlated at channel 18 (*r* = 0.547, *p* < 0.05, two-tailed). No significant correlation was found between honesty INS and eye contact; (**A**) the female dyads showed a significant correlation of deception INS and times of eye contact at channel 18 (*p* < 0.01, two-tailed). (**B**) The male dyads showed no significant correlation of deception INS and times of eye contact at any channels. [Color bar represents r value].

To assess the relation between INS and behavior, Pearson correlation analyses were conducted on the coherence value at all channels and on eye contact from each dyads. In all dyads, the deception INS and eye contact were positively correlated at channel 18 (*r* = 0.547, *p* < 0.05, two-tailed). No significant correlation was found between honesty INS and eye contact, indicating that the INS was associated with deception behavior. Separate analyses for both genders between deception INS and eye contact revealed that only the female dyads showed a significant correlation at channel 18 (*r* = 0.803, *p* < 0.01, two-tailed). This relationship was not significant in male dyads. No significant correlation was found at any other channels (See in Fig. 4).

#### Directional Coupling

**Figure 5:**
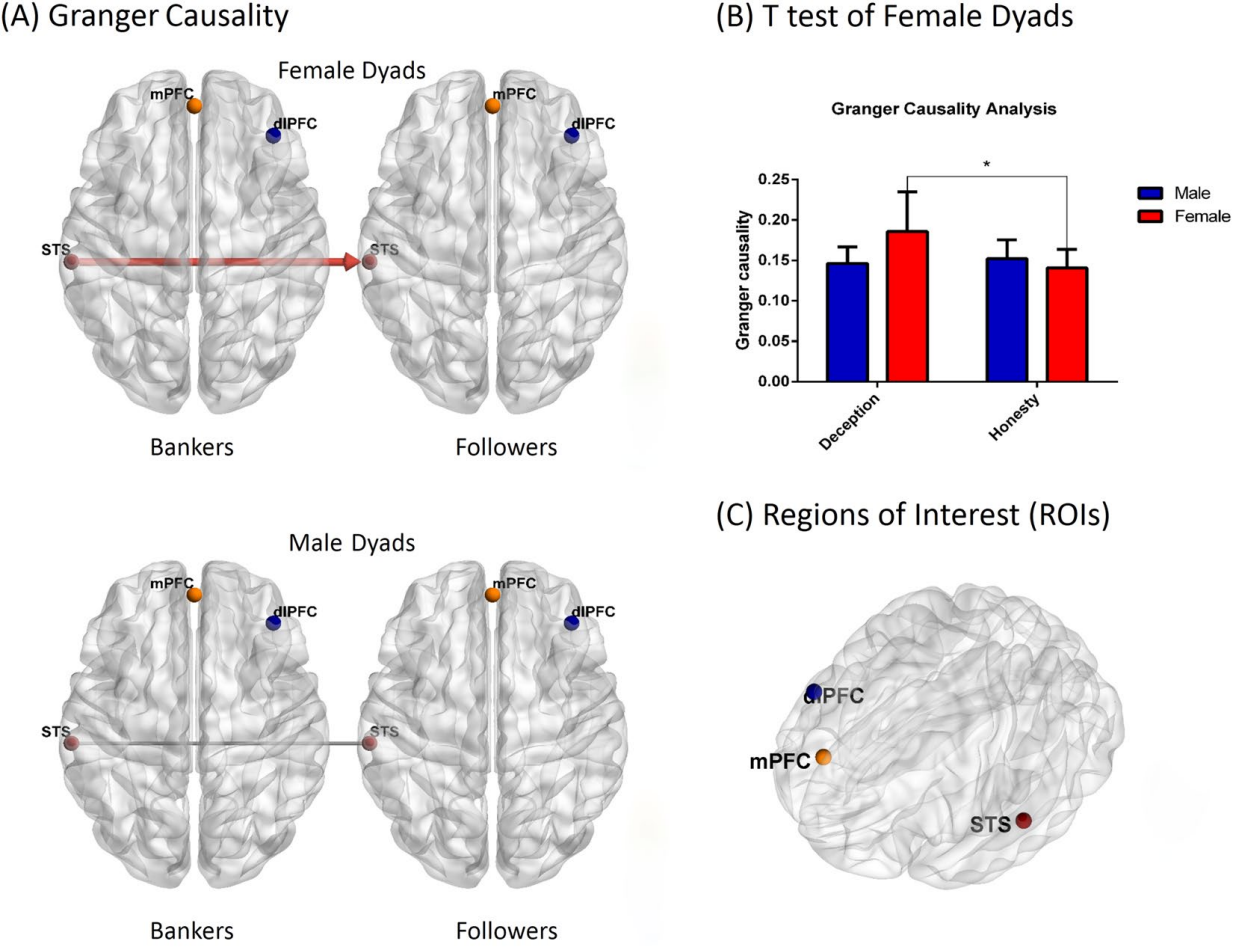
Directional coupling in female and male dyads. (**A**) The G-causality was analyzed in two directions (from bankers to followers, from followers to bankers). Direction was indicated by arrow and Significant G-causality was indicated by a red solid-line (*P < 0.05, FDR corrected) (**B**) Mean G-causalities in two conditions and two genders illustrated in the bar graphs. Only in female dyads, the mean causality from bankers to followers (indicated by red color) was significantly higher in the deception conditions than in the honesty conditions (*P < 0.05). Error bars indicate standard errors (FDR corrected). (**C**) Regions of interest in this research. mPFC, medial prefrontal cortex; dlPFC, dorsolateral prefrontal cortex; STS, superior temporal sulcus. [All 3-D brain figure was edited based on *BrainNet Viewer* (Xia, 2013)].

To determine the direction of INS at TPJ and pSTS (channels 16, 17, 18, 19) we conducted a 2 × 2 ANOVA (in all channels) with gender (male vs. female) as a between-participant factor and behavior-type (deception vs. honesty) as a within-participant factor, and mean Granger causality as the dependent variable. For the bankers-to-followers direction, the ANOVA result revealed a main effect of behavior-type in channel 19 [*F*(1,16) = 6.428, *p* = 0.022, ${\eta }_{p}^{2}$ = 0.287, (FDR corrected)] and a significant interaction effect [*F*(1,16) = 10.824, *p* = 0.005, ${\eta }_{p}^{2}$ = 0.404]. A simple effect test found that in the female dyads the mean G-causality of deception was significantly larger than honesty [*t*(8) = 3.107, *p* = 0.030, d = 2.197, Bonferroni adjusted], but these difference did not exist in male dyads [*t*(8) = −1.084, *p* = 0.310, d = −0.767]. A significant gender effect was not detected in the condition of deception [*t*(8) = 2.147, *p* = 0.064, d = 1.518] or honesty [*t*(8) = −1.163, *p* = 0.279, d = −0.822]. No gender effect was found in the bankers-to-followers directions. Furthermore, no significant G-causality differences were found in other channels or in the followers-to-bankers direction (*p*s > 0.05) (See in Fig. 5).

## Discussion

The purpose of the present study was two-fold: First, we aimed to examine the neural mechanisms underlying spontaneous deception in face-to-face interactions from a perspective of two-person neuroscience^48^. Second, we explored potential gender differences. To achieve these goals, we simultaneously measured pairs of participants’ fronto-temporal activations in a turn-based, gambling card-game using an fNIRS hyperscanning system. To our knowledge, this was the first such attempt to investigate inter-brain correlates of deception in real face-to-face interactions^49^.

The behavioral results demonstrated that deception was a common tactic across conditions of the card task. However, results did not reveal differences in the deception rate between male and female players. This goes against previous research that had reported that males often generate more deception than females, especially in academic dishonesty studies (see details in the introduction section)^42, 43, 45, 50^. On the other hand, our finding is in keeping with several other studies which have not shown a gender effect. For instance, Cooper and Peterson (1980) reported that cheating could be explicitly encouraged by another person but with no gender effect^51^. Fisher and Brunell (2014) directly examined gender effects in three cheating conditions: (1) with imposed pressure to be honest (participants thought they were being monitored by a lie detector); (2) an anonymous condition; and (3) a public condition in which participants were instructed that their responses would be reviewed by others. Results demonstrated that the gender effect disappeared in the public condition^52^. Thus, the gender effect on cheating behaviors may largely depend on the “social” feature of the tasks, which may explain the present behavioral data and the importance of more ecologically valid studies.

On the other hand, while basic deception use was undifferentiated, other behavior aspects—specifically eye contact—did show significant gender difference. Consistent with previous findings, our results showed that female dyads generated more eye contact than males. One supporting hypothesis is that females are more likely to understand others in the presence of visual contact, but males do not, or may use contact more as a special threat^53–56^. The eye contact differences also emerged mostly in the banker betting periods, where the deception behavior was specifically expected to occur. Compared with other periods, subjects may have needed to acquire valuable information from each other in this period—e.g., a follower needed to guess which card the banker received or if they had been cheated—presumably leeding to need for such extra interpersonal information.

With respect to the fNIRS data, there were two main findings relating to both the neural correlates of spontaneous deception and to gender effect. First, analysis revealed higher activation in deception than in honesty in the TPJ (channel 16 and 17) during the banker betting period in the banker’s brain. The inter-brain analysis of both participants further demonstrated that, compared with the honesty situation, the banker-follower pairs showed positive inter-brain coherence in the left pSTS (channel 18 and 19) in the deception situation. Previous findings have suggested that the location of cortical activation and inter-brain coherence do not necessarily coincide. Conversely, the present findings highlight the utility of assessing multiple aspects of fNIRS hyperscanning data when investigating social cognition^57^. Recent study by Tang (2016) has also revealed an interpersonal neural synchronization in rTPJ during naturally occurring face to face economic exchange^58^. Research has reported that the pSTS is involved in the ‘social brain network’ aspect of theory-of-mind (ToM)^59–61^. The pSTS is associated with joint attention, generating shared focus on an object across individuals for understanding, and predicting others’ actions and intentions^62–65^. The TPJ plays a unique and independent role in processing social information about future behavior^66^. Thus, both the intra- and inter-brain results suggest that spontaneous deception in face-to-face interactions may involve more social processes of mentalizing. Such deeper involvement of mentalizing may also be the reason for more eye contact in deception periods, also supported by our finding of higher activation and INS of the TPJ in betting periods. Previous studies also highlight joint attention in TPJ^67, 68^, which indicates an alignment between joint attention and the interpersonal neural synchronization in this region. However, our GCA (granger causality analysis) results showed that the direction of INS from bankers to followers in the deception condition was stronger than in the honesty condition, implying that the primary information flow was from bankers to followers. This is interesting in that, if one only takes the joint attention function into account, bankers may generated more eye contacts than followers, which is inconsistent with what might be expected in deception (i.e., a deceiver might be expected to avoid eye contact). The GCA results, on the other hand, are consistent with previous dyadic studies—brain activity in a gesturer may predict that of a guesser^69^; brain activity of a model may predict that of an imitator^70^; or a leader’s activity may be more important in predicting results when compared to followers^32^. In our study, bankers led the activity and affected the decision-making of followers, leading to stronger effects in the deception condition. These results suggest that TPJ and pSTS may be involved in complex interactive movements and dyadic communication in social cognition.

Concerning the gender effect, the intra-brain data revealed no activation differences between males and females in all three periods. By contrast, the INS-Behavior relation results and the inter-brain results revealed that only females showed positive significant inter-brain coherence in the TPJ in deception situations compared to honesty situations. Our GCA results further showed that only in the female dyads was the direction of INS from bankers to followers in the deception condition stronger than in honesty conditions. This implies that, again only in female dyads, deception behavior may elicit a stronger primary information flow from bankers to followers. A recent study by Tang^58^ also observed significantly increased inter-brain coherence in the temporal cortex for female-female dyads during a simultaneous key-pressing task^34^. They offered the explanation that female players relied on action-centered social cognitive processes during cooperation, with this cognitive strategy between females positively influencing cooperation performance. Similarly, our INS-Behavior relation results revealed positive correlation of deception INS with female dyads’ eye contact (however, see also Pan (2017) who did find increased inter-brain coherence in right superior frontal cortex (rSFC) for male-female lover dyads^36^). Compared with the previous two studies, our research of course focused on deceptive behavior related to competition rather than on cooperation. Thus the present differences may be a result of experimental design. In addition, in Pan and colleagues’ study the two participants in a dyad sat side-by-side, separated by a board, while in our and Baker’s studies, dyads sat face-to-face. Thus, we assume that the similar pattern of inter-brain results may also stem from the face-to-face mode. The present study, from a perspective of two-person neuroscience, also supports the social nature of females and suggests that females may utilize more mentalizing processes to deduce another person’s thoughts and beliefs when they try to make a deceptive decision, showing inter-brain synchronization in the TPJ.

It is also noteworthy that the present study did not find evidence for significant activation patterns in the DLPFC and mPFC in both deception and honesty conditions, regardless of gender. This is not consistent with some previous findings that deception requires higher activations in the DLPFC^39, 71–74^. The DLPFC is associated with cognitive functions such as executive control and behavioral inhibition^71, 75–77^. In the previous studies using paradigms of compulsive deception, participants needed to inhibit their responses of truth-telling in the deception conditions, but no cognitive load was imposed in the honesty conditions. Whereas in our study, participants generated deception or honesty behavior spontaneously, depending on their own strategy to win the monetary reward. Thus, both the deception and honesty situations may have required executive control involving the DLPFC. Similarly, while the mPFC is thought to be part of the “theory of mind” (ToM) brain network, which is activated by considering the intentions of another individual in social processing^78, 79^, in our study this may have been recruited in all conditions. The natural environmental setting may have also led to different activations, or our sample may have been insufficiently large to detect subtle differences. See also Tang^34^ for similar lack of mPFC evidence.

The present study of course also had additional limitations and areas in need of future research. First, issues relate to the ecological validity of the gambling game. Although we obtained significant results on deception through the classification of the bets, in real social economic encounters the monetary reward/risk involved in deceptive behaviors is often much larger, which may change individual’s decision-making processes. The ecological validity of the present study still cannot completely coincide with complex, daily-life economic interactions that may lead to our choice to deceive. In spite of that, the present study does move an important step forward to the understanding of daily-life cognition. Second, the present study adopted a relatively real card-game, making it difficult to collect other behavioral evidence such as reaction time (RT). Further study is needed to optimize the game paradigms to collect more behavioral indices.

A third limitation concerns motion artifacts. Our experiment controlled participants’ head movements and other unrelated actions. However, the card-game itself required body movements, which may have induced extra noise and in turn reduced the quality of the fNIRS data. Although fNIRS is tolerant to body movements, it is important to be mindful of this limitation when making inferences. Last, the sample size for comparison between male and female participants (i.e., gender effect) was rather minimal. Future study is needed to confirm the present gender effect results. That said, this study revealed neural differences in dyads inter-brain coupling, and hopefully will lead the way to much further insight on this topic.

## Methods

### Participants

Sixty university students (26 males, 22.3 ± 2.4 years old, all native Chinese speaking) participated in the experiment. All participants were right-handed, as assessed via questionnaire, and had normal or corrected-to-normal vision. Participants did not know each other before the experiment and were randomly paired in same-gender dyads to avoid cross-gender effects^80^. They were informed about the purpose of the experiment, and written informed consent was obtained prior to participation. All methods were carried out in accordance with the guidelines of the Declaration of Helsinki, and all experimental protocols were approved by Institutional Review Boards of Southeast University.

### Experimental procedure

**Figure 6:**
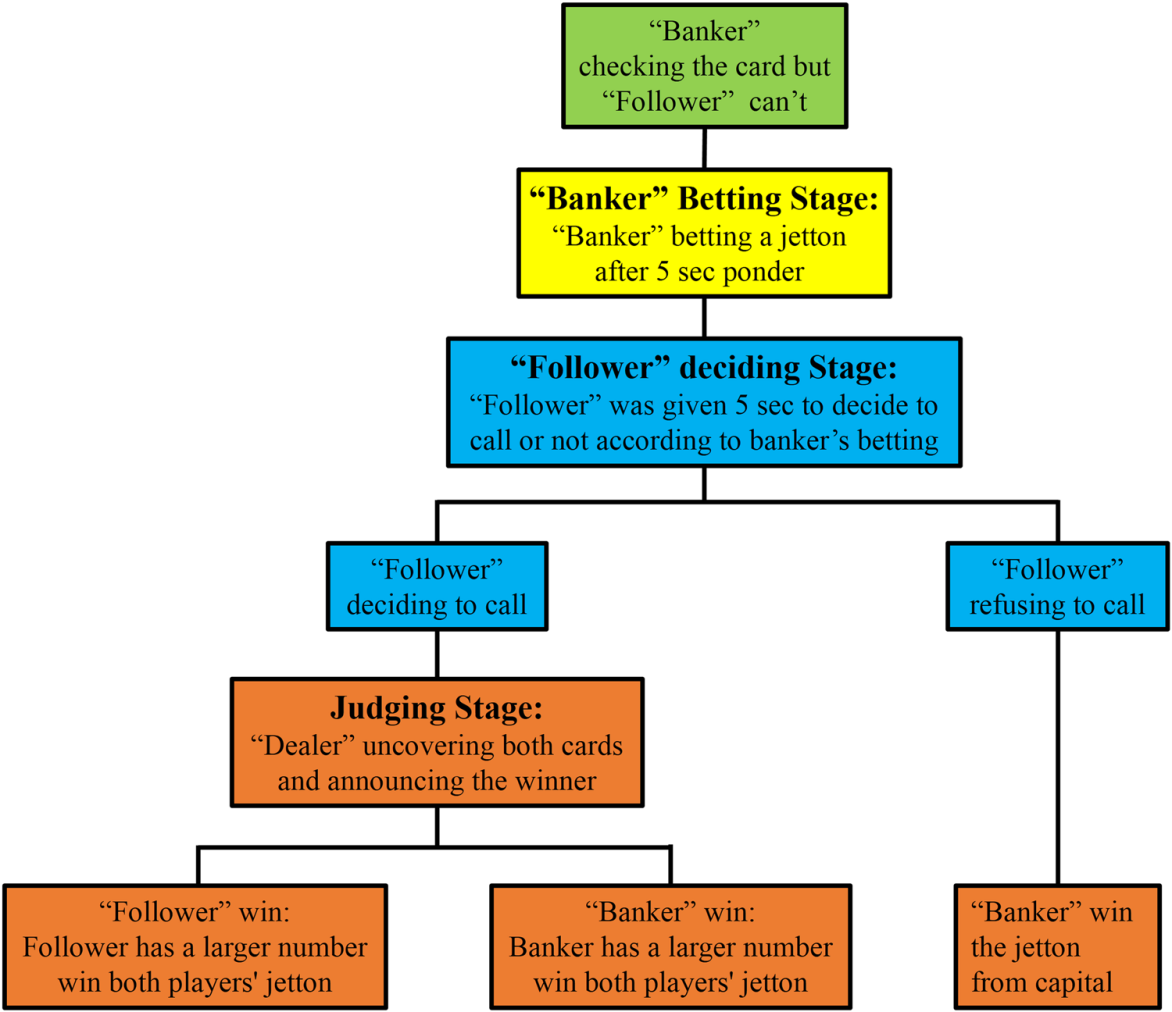
Procedure of each round of the card game. In each round, the dyads were randomly given one card per participant. The participant designated as the “banker” was then instructed to check his/her card and bet a jetton (1, 3, or 5 RMB) after 5 sec in which the banker could generate his/her decision. The other participant, the “follower,” was then given 5 sec to decide to call or not according to banker’s betting without checking his/her own card. If the follower refused to call, the banker won the jetton. In contrast, if the follower decided to call, the dealer would uncover both cards and announced the winner (the participant whose card had a larger number won both players’ jetton). The dealer would then re-shuffle all five cards ending the round. The banker in the first round was chosen by a Finger Guessing Game, with banker-follower roles then alternating in every subsequent round. Participants were prohibited from discussing during the experiment.

Figure 6 illustrates the experimental procedure (See in Fig. 6). The present study used a two-person card-game. Two participants, sitting face-to-face, took different roles and played the game in a turn-taking style^29^. The roles consisted of a ‘banker’ who led the betting and who had the option of deceiving their opponent and a ‘follower’ who responded to their opponent’s bet and could determine if they were being deceived. The experimenter acted as a dealer throughout the game. The banker for the first round was chosen by a finger guessing game. The roles were then switched for each subsequent round of the game.

Prior to the experiment, each participant was given the equivalent of 50 renminbi (RMB; ~7.5 US Dollars) in chips from the experimenter/dealer as capital. Chips were divided into three types: 1, 3 or 5 RMB. Participants were told that this money was not automatically theirs to keep. Rather, they should use this capital to win money for themselves within the game. The whole experiment consisted of 30 rounds of the card game. After finishing all rounds, any money won by each participant was given to them as remuneration.

The card game followed a modified “stud poker” style, with participants allowed to bet and then to reveal their cards (normal playing cards with the printed number of 3, 4, 5, 6, or 7, composing a five card deck), and with the highest card winning the game. In each round, both participants were each given one card, face down, drawn randomly from the deck. The ‘banker’ was then allowed to view his/her card and to place a bet, using only one of the three chip denominations, within five seconds. The ‘follower’ was not allowed to look at their card and was then given five seconds to decide if they would “call” the banker (i.e., match their bet). If the banker was called, both participants revealed their cards, with the highest card the winner and the corresponding participant pocketing both bets for themselves. If the follower did not call, the banker automatically won, and was allowed to pocket their original bet—i.e., moving the money from capital into their own winnings. Following each round, the dealer re-shuffled the deck. Participants were given several practical rounds to understand the game protocol and were prohibited from conversing verbally during the experiment.

This design therefore placed an incentive on the banker to use either deception or no deception, depending on the wager and their drawn card, and for the follower to try to assess whether they were being deceived when making a matching call. For example, a low rank card in the banker’s hand would have a lower winning chance. Thus, it was in the banker’s best interest to either: (1) minimize their loss by using a corresponding low bet (the 1 RMB chip), while also risking being called and losing; or (2) persuade their partner not to call by using a high bet, and pocketing their side of the wager. On the other hand, if the banker had a high card, they could either maximize their winnings by using a high chip, hoping that the other player would either not call, giving them 3 RMB, or (falsely) consider them to be attempting deception and call, potentially doubling the banker’s winnings. Transversely, the banker could use a lower bet, and hope to increase the odds that their partner would call.

### NIRS Apparatus

**Figure 7:**
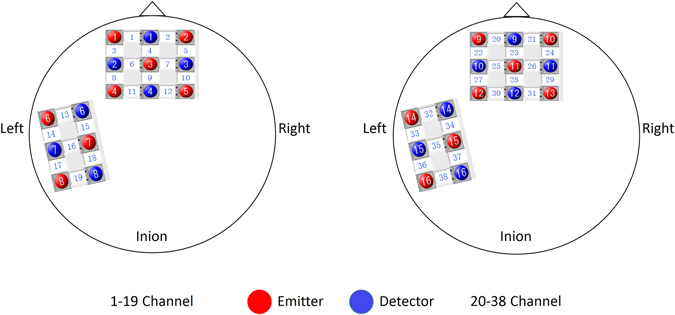
Positions of fNIRS channels. Nine optodes (five emitters and four detectors) were attached to the forehead in a 3 × 3 lattice pattern forming 12 measurement channels. The distance between adjacent emitters and detectors was 3 cm, and the central area between a paired emitter and detector was defined as a measurement channel. The leftmost channel in the lowest line was located at the center position of Fp1 and Fp2 of the international 10–20 system. Accordingly, the frontal panel covered approximately the medial prefrontal cortex (mPFC) and the dorsal lateral prefrontal cortex (dlPFC). The remaining six optodes (three emitters and three detectors) were placed on the left temporal lobe in a 2 × 3 lattice pattern forming 7 measurement channels. The middle channel in the lowest line was located at the T3 position of the international 10–20 system, and thus the left panel covered approximately the posterior superior temporal sulcus (pSTS).

To simultaneously measure brain activity of both participants, we used a 30-channel fNIRS system (LABNIRS; Shimadzu Co., Japan) operated at 780, 805, and 830 nm wavelengths, which could detect relative concentration changes of oxygenated hemoglobin (Oxy-Hb), deoxygenated hemoglobin (Deoxy-Hb), and total hemoglobin, assessed via the conversion of light intensity signals using the modifed Beer-Lambert law^81^, all proven measures of relative brain activation^82^. Each participant was assigned 15 optodes. Nine optodes per participant (five emitters and four detectors) were attached to the forehead in a 3 × 3 lattice pattern forming 12 measurement channels. The distance between adjacent emitter and detector pairs was 3 cm, with the center point between pairs defined as the measurement channel. As Fig. 7 illustrates, the leftmost channel in the lowest row was located at the center position of Fp1 and Fp2 following the international 10–20 system for EEG. Accordingly, the 3 × 3 lattice covered the medial prefrontal cortex (mPFC) and the dorsal lateral prefrontal cortex (dlPFC). The remaining six optodes per participant (three emitters and three detectors) were placed over the left temporal lobe in a 2 × 3 lattice pattern, forming 7 measurement channels. The middle channel in the lowest row was located at the T3 position of the international 10–20 system, and thus the lattice covered the posterior superior temporal sulcus (pSTS) (See in Fig. 7). The sampling rate was 57 Hz.

Before the first round, a 30-sec rest was given as a baseline of participant’s brain activity. The inter-game-interval was 10 seconds, and all 30 rounds of the game lasted about 30 min. The time-course of the experiment was controlled by voice prompts generated via an E-PRIME program.

### Data analysis

#### Behavioral results: deception vs. no deception

To assess whether the banker had attempted to deceive their opponent, both cards and betting chips were divided into three ranks. Among the potential wagers, a 1 RMB token was ranked lowest, a 3 RMB had a middle rank, and a 5 RMB was considered as the highest rank. Similarly, of the five cards (3, 4, 5, 6, 7), those with numbers ‘3’ and ‘4’ were given the lowest rank, corresponding to the odds of the banker winning if their bet was called; ‘5’ belonged to the middle rank; ‘6’ and ‘7’ were considered as the highest rank. Deception was identified when the banker’s card rank was inconsistent with his/her wager rank (see also description in Experimental Procedure above).

#### NIRS data

Data obtained from one pair of participants was excluded due to machine malfunction. The remaining 29 pairs were used in the analysis. Matlab 2012a was used to process the data, and statistical analyses were conducted by Statistical Package for the Social Sciences (SPSS). The significance level was set at *p* < 0.05. False discovery rate (FDR) correction was used to minimize the multiple comparison problem. Cohen’s d for only one channel one sample t-tests was also calculated. Bonferroni adjusted p-values were used for all pairwise comparisons.

We focused our analysis on Oxy-Hb, since this has been shown to be the most sensitive signal to changes in cerebral blood flow, out of the three signals detected by the NIRS apparatus^83, 84^. In order to remove motion artifacts and physiological noise from the raw data, a band-pass filter (0.01–0.2 Hz) was first applied to the raw data. This was followed by a linear baseline correction, using the 30-sec rest period before all rounds as the baseline, and a Z-score transformation^85–87^. The data for each of the 30 rounds was then divided into two groups (deception vs. honesty), based on the above analysis of banker’s decisions. Finally, the group-averaged data was obtained.

#### INS (interpersonal synchronization)

For the analysis of synchronization between two players’ fNIRS data, MATLABVC package of Wavelet Transform Coherence (WTC) (http://noc.ac.uk/usingscience/crosswavelet-wavelet-coherence)^88^ was used to calculate the relationships between the NIRS signals generated from banker-follower dyads. Wavelet coherence measures the correlation between two signals’ component on both frequency and time, and has been successfully applied in several fNIRS hyperscanning studies^32, 36, 37, 58, 89^. FNIRS Oxy-Hb time series from the banker betting stage to judging stage (i.e. one complete experiment trial) were used to calculate the interpersonal brain coherence as synchronization between pairs. We identified a frequency band between 2 s and 7 s (between0.05 and 0.2 Hz), where the task occurred and also remove high and low frequency noise. We calculated the average coherence value in this band during the two task blocks. “Coherence increase” is defined as the average coherence value in the deception blocks, minus the honesty blocks. We then performed a one-sample t-test of “coherence increase” for each channel across all participant dyads.

#### Directional coupling

Granger causality uses vector autoregressive models to measure the causal relationship between time series in brain data, and has been successfully used in estimation of directional coupling between cortical areas using Near-Infrared Spectroscopy^36^. A Granger Causality tool was used to analyze the interdependence of synchronization for all channels (see more details in http://hermes.ctb.upm.es/). Oxy-Hb signals during the task periods were divided into deception and honesty sequences and the pairwise conditional Granger-causality (G-causality) of both participant directions were calculated (i.e., from bankers to followers; from followers to bankers). A 2 × 2 ANOVA (in all channels) was then calculated with gender (male vs. female) as a between-participant factor and the behavior-type (deception vs. honesty) as a within-participant factor.
